# Dermatopathology 2030: Navigating the Digital, Computational and Molecular Convergence for Precision Diagnostics—Originated from a Round Table Discussion at A 66, Basel

**DOI:** 10.3390/dermatopathology13040047

**Published:** 2026-10-02

**Authors:** Carlo Tomasini, Maria-Teresa Fernández-Figueras, Gürkan Kaya

**Affiliations:** 1 Dermatologic Clinic, Department of Clinical-Surgical, Diagnostic, and Pediatric Science, University of Pavia, Fondazione IRCCS Policlinico San Matteo, 27100 Pavia, Italy; 2 Area de Diagnòstic Anatomopatològic, Hospital Universitari General de Catalunya, Grupo Quirónsalud, Universitat Internacional de Catalunya, 08017 Barcelona, Spain; maiteffig@gmail.com; 3 Departments of Dermatology and Clinical Pathology, University Hospital of Geneva, 1205 Geneva, Switzerland

**Keywords:** dermatopathology, digital pathology, whole slide imaging, artificial intelligence, Next-Generation Sequencing (NGS), Gene Expression Profiling (GEP), Spitz melanoma, OPTN::RET fusion, spatial transcriptomics, precision medicine, clinicopathologic correlation, diagnostic reasoning, diagnostic safety

## Abstract

**Background**: Traditional dermatopathology relies on analog, qualitative pattern recognition, which encounters critical limitations when facing diagnostic “gray zones” and ambiguous tumor proliferations. **Objectives**: This paper synthesizes a comprehensive framework integrating Whole Slide Imaging (WSI), Artificial Intelligence (AI), and multi-omics to evaluate how this structural convergence redesigns diagnostic risk and safety ecosystems toward 2030. **Methods**: We analyze the conceptual shift from individual microscope-based observation to multiple data integration. We integrate recent algorithmic computational breakthroughs—including virtual histological staining, deep learning margin mapping, and multimodal report generation—and benchmark human inter-observer discordance. These dimensions are contextualized through an index adult case study from the University Hospital of Geneva. **Results**: Clinical digitization converts qualitative tissue images into quantitative datasets, potentially reducing some sources of individual cognitive error while introducing new systemic vulnerabilities. The clinical necessity of multi-omics is driven by the limitations inherent in human interpretation of morphology. In our index clinical case of a 45-year-old male with a right arm lesion mimicking a seborrheic keratosis, standard morphology and a wide immunohistochemical panel established a diagnosis of melanoma. High-throughput next-generation sequencing (NGS) provided the molecular evidence to refine the biological classification of the tumor through identification of an oncogenic OPTN::RET fusion, supporting a diagnosis of Spitz melanoma according to the 5th edition of the WHO classification. Layering bulk Gene Expression Profiling (GEP) provides an emerging adjunctive prognostic tool to stratify 5-year recurrence risk and manage clinical crossroads regarding Sentinel Lymph Node Biopsy (SLNB) concordance. **Conclusions**: There is little doubt that AI will improve histopathological workflows, but its potential weaknesses make human supervision mandatory. By the next decade, dermatopathologists must evolve into multimodal biological experts, responsible for integrating morphology, molecular biology, clinical information, and artificial intelligence. Technology may scale information across global networks, but diagnostic safety should continue to depend on human judgment, contextual interpretation, and the ability to navigate uncertainty.

## Simple Summary

This manuscript charts the transformation of dermatopathology toward 2030 through the convergence of whole slide imaging, artificial intelligence, and advanced molecular diagnostics. Using an ambiguous OPTN::RET fusion-positive Spitz melanoma as an index case and recent evidence of human diagnostic discordance, we illustrate how the field is shifting from qualitative pattern recognition toward high-dimensional biological data integration. Diagnostic safety increasingly depends on the integration of morphology, genomics, computational intelligence, and clinical reasoning within interconnected diagnostic networks.

## 1. Introduction: The Decline of Pure Analogue Pattern Recognition

For over a century, the optical microscope has been the undisputed cornerstone of dermatopathology. For generations of pathologists, diagnostic authority has been based on an extremely refined, yet inherently subjective, process of qualitative and analogue recognition of morphological patterns. Pathologists have trained their eyes to recognize cellular architecture, the staining properties of histochemical stains and the spatial arrangement of cell populations within skin tissue. Although this independent visual approach allows for the successful classification of the vast majority of routine dermatological conditions, it encounters a substantial limitation when confronted with so-called biological ‘grey zones’ and borderline melanocytic proliferations.

As emerged during the international roundtable organized by the European Society of Dermatopathology and MDPI, *Dermatopathology 2030: Navigating the Digital and Molecular Frontier*, our discipline is undergoing a profound and irreversible paradigm shift. Rather than relying on isolated individual observation, dermatopathology is evolving into a high-dimensional ecosystem in which digital infrastructures, computer vision algorithms and advanced molecular biology techniques converge. This transition is already changing the day-to-day practice of dermatopathology and will have an increasingly profound impact on diagnostic pathways over the next decade. By rethinking the way we acquire, process and interpret tissue information, we can ensure that dermatopathology continues to serve as the benchmark for decision-making in personalized medicine.

The year 2030 should not be interpreted as a specific target date, but rather as a conceptual horizon within which the current convergence of digital pathology, artificial intelligence and molecular diagnostics is expected to redefine the professional identity of the dermatopathologist.

## 2. Whole Slide Imaging: From Digital Slides to Adaptive Diagnostic Ecosystems

Whole Slide Imaging (WSI) represents the technological foundation of digital transformation in pathology. By replacing the physical glass slide with high-resolution digital images that can be easily archived, shared, and analyzed, WSI overcomes many of the limitations of conventional microscopy. This transition has been driven primarily by the need to overcome the logistical limitations of traditional glass slides, improve workload distribution, facilitate multidisciplinary collaboration and remote specialist consultation, and enable the integration of artificial intelligence (AI) into routine diagnostic practice, where it acts as an active cognitive mediator, transforming raw data into structured information.

The transition from conventional to digital pathology extends well beyond slide digitization. It requires a comprehensive redesign of the entire diagnostic workflow, including laboratory organization, sample preparation, quality control procedures, IT infrastructure, staff training, and data management. More importantly, as pathology laboratories become increasingly interconnected, diagnostic performance no longer depends solely on the expertise of the individual pathologist but also on the reliability of the entire digital ecosystem. This ecosystem encompasses image acquisition, data integrity, interoperability between systems, algorithmic support, and the effective integration and communication of clinical information [1].

**Figure 1:**
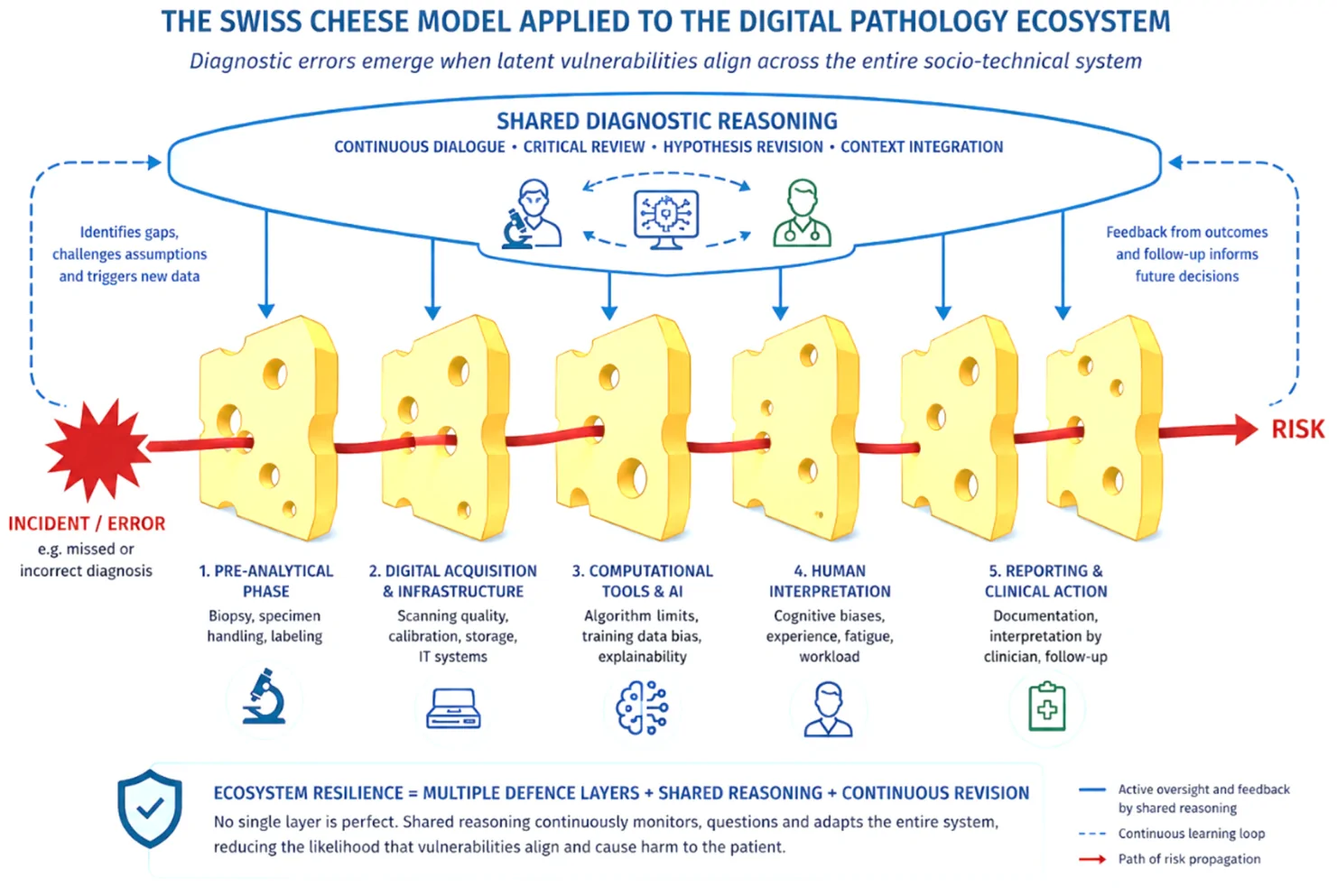
Swiss Cheese Model adapted to the digital pathology diagnostic ecosystem. The model illustrates how vulnerabilities across multiple diagnostic layers may align and lead to error, while shared diagnostic reasoning acts as a continuous cross-cutting process supporting system resilience. This figure was created with the assistance of generative artificial intelligence (OpenAI ChatGPT, accessed in 2026; model version not recorded) based on the authors’ original scientific concept and design. The authors critically reviewed, modified, and approved the final version and assume full responsibility for its scientific content and interpretation.

In this socio-technical ecosystem, humans, digital infrastructures, AI, molecular diagnostics, and clinical information continuously interact. Consequently, digital slides, raw image data, computational algorithms, and network infrastructures are not passive technological components but active cognitive mediators that encode, integrate, and transform diagnostic information. Diagnosis becomes an integrative process in which diagnostic confidence increasingly depends on the coherent integration of multiple complementary data streams. The redistribution of the diagnostic process across this interconnected socio-technical ecosystem inevitably reshapes the distribution of clinical risk. In this context, James Reason’s Swiss Cheese Model offers a useful conceptual framework for understanding how digital pathology reshapes clinical risk [2]. According to this model, diagnostic errors (incidents) emerge not only from human mistakes, but from the alignment of latent vulnerabilities within the entire diagnostic ecosystem (Figure 1). Consequently, the resilience of this digital diagnostic ecosystem—and consequently patient safety—depends not only on the robustness of its technological infrastructure but also on the way diagnostic reasoning is generated, shared and continuously revised across its human and computational components.

Each diagnostic layer contributes to preventing error, but also contains potential vulnerabilities. Diagnostic incidents occur when weaknesses across multiple layers align. Unlike the original model, shared diagnostic reasoning is represented as a continuous, cross-cutting cognitive process that monitors, challenges and revises diagnostic hypotheses across the entire workflow, strengthening system resilience and reducing the likelihood of error propagation.

## 3. Shared Reasoning: The Cognitive Layer of Digital Pathology

Within this distributed diagnostic ecosystem, the central challenge is no longer simply the acquisition or processing of information, but the construction of reliable diagnostic reasoning. Whilst distributed reasoning enables information to circulate across the digital ecosystem, shared reasoning enables that information to be critically interpreted, challenged, and transformed into reliable diagnostic knowledge [3]. Shared reasoning therefore functions as a cognitive safety barrier, continuously testing diagnostic hypotheses against new clinical, morphological, and molecular evidence, thereby reducing the risk of premature diagnostic closure. Shared reasoning can be viewed as the cognitive layer that transforms distributed information into shared diagnostic knowledge through collaborative abductive inference.

**Figure 2:**
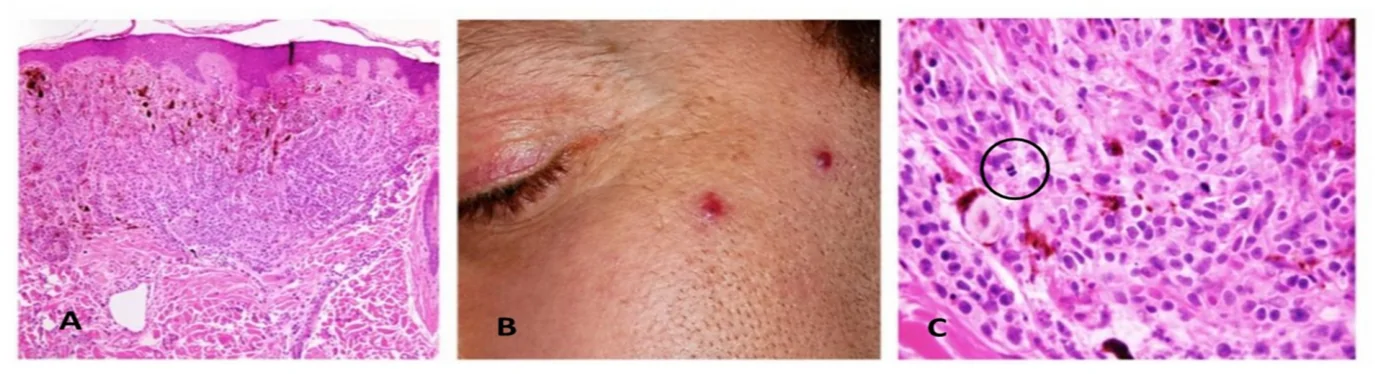
(**A**) Low-power view showing a dermal melanocytic proliferation initially interpreted as a benign dermal naevus (40×). (**B**) Check of the patient revealed a history of a previous invasive melanoma, prompting diagnostic re-evaluation. (**C**) High-power re-examination of the lesion demonstrating a subtle dermal mitotic figure (circle), previously overlooked during the initial assessment (400×). The final diagnosis was dermal metastases of melanoma.

This dynamic process becomes particularly evident when different sources of information appear inconsistent. It is precisely the discrepancy that triggers a second level of diagnostic reasoning. For example, a lesion initially interpreted as a benign dermal naevus may be completely re-evaluated at high magnification when the clinical information of a previous invasive melanoma emerges, leading to the identification of subtle dermal mitoses (Figure 2).

**Figure 3:**
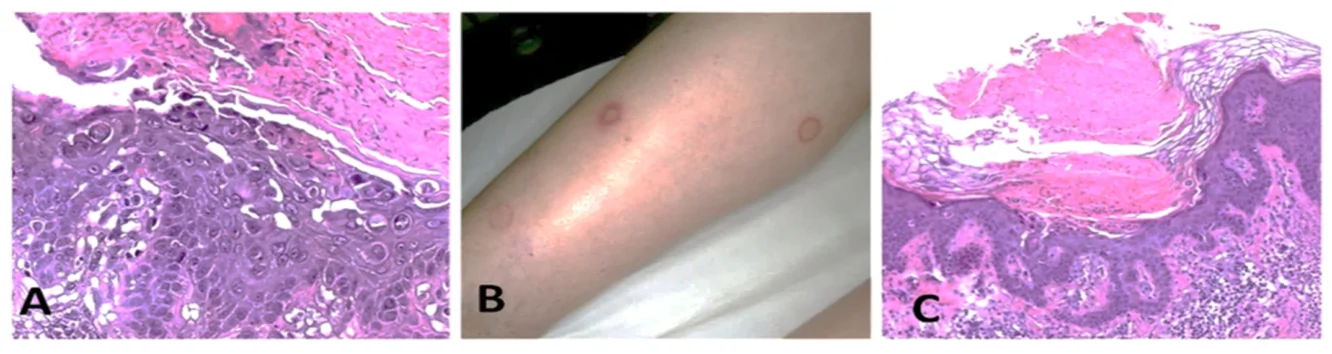
A pattern initially suggestive of Darier disease (**A**, 400×) was ultimately reinterpreted as porokeratosis with Darier-like dyskeratosis (**C**) after clinicopathological correlation (**B**, 100×).

Pattern recognition naturally privileges the most immediately available explanation. However, when morphological findings conflict with the clinical presentation, shared reasoning requires the observer to suspend the initial interpretation and actively search for alternative explanations. Figure 3 illustrates this process.

Shared reasoning not only improves diagnostic consistency but also creates the cognitive conditions necessary for abductive reasoning. When no existing diagnostic category adequately explains the available evidence, the observer must generate new explanatory hypotheses capable of reconciling apparently conflicting findings.

In complex diagnostic systems, abductive reasoning emerges from the integration of multiple sources of evidence, including histopathological findings, clinical information, molecular data and multidisciplinary discussion. Artificial intelligence can support this process by identifying patterns and ranking diagnostic hypotheses. However, when the available evidence remains incomplete or internally inconsistent, generating new explanatory hypotheses still relies largely on human reasoning. This ability depends on continuously integrating new information, re-evaluating previous interpretations and exploring alternative explanations throughout the diagnostic process. This cognitive flexibility—the capacity to revise hypotheses as new evidence becomes available—remains one of the defining strengths of human diagnostic reasoning. Although AI can efficiently analyze large amounts of multimodal information, it currently lacks the cognitive flexibility required to actively reshape the diagnostic problem by seeking new contextual information or reformulating explanatory hypotheses when existing categories fail.

In sum, distributed reasoning provides access to heterogeneous sources of information; shared reasoning critically integrates them into a coherent interpretation; and abductive reasoning becomes necessary whenever this integrated evidence still fails to explain the diagnostic problem. Future dermatopathology will therefore depend not only on increasingly sophisticated digital infrastructures but also on preserving the cognitive openness required for reliable diagnostic reasoning. AI and WSI should augment, rather than constrain, the clinician’s ability to explore alternative hypotheses, integrate new evidence and maintain traceable diagnostic reasoning throughout the decision-making process.

## 4. Software as the Co-Pilot: Computational Pathology and Artificial Intelligence

The infrastructure established by widespread histopathological digitalization serves as the mandatory launching pad for computational pathology. Once an analog glass slide is scanned, its qualitative optical properties are converted into a structured, quantitative matrix of pixel-level and tissue-level features. This enables the mathematical interrogation of color profiles, textural patterns, cellular shapes and spatial topology.

The clinical suitability for AI in dermatopathology is driven by three main factors: high visual overload causing mental fatigue, the need to document tedious parameters in standardized reports, and the undeniable inter-observer discordance in some difficult cases [4].

To harmonize this diagnostic variance, computational workflows are deploying targeted deep learning applications. In a landmark study, Löper et al. demonstrated automated surgical margin assessment for lentigo maligna via a Sox-10-based melanocyte density mapping algorithm [5]. The system generates automated, color-coded heatmaps that segment melanocyte density into clear risk zones: green signals indicate low density (<25 melanocytes/0.5 mm), yellow indicates a borderline zone (25–29 melanocytes/0.5 mm), and red flags a high-density area (≥30 melanocytes/0.5 mm) associated with positive surgical margins. Concurrently, deep learning models have achieved virtual histological staining, computationally translating a standard bright-field H&E image into a virtual Melan-A or Sox-10 immunohistochemical stain in seconds, bypassing physical lab processing [6].

Furthermore, the rise of multimodal foundation models is redefining clinical synthesis by combining data from multiple sources—such as gigapixel images, medical texts, and 3D scans—into a single, accurate diagnostic report. Tran et al. introduced HistoGPT, a vision–language transformer model trained on millions of biomedical articles, capable of generating comprehensive, human-grade diagnostic reports directly from gigapixel whole slide images for common, homogeneous malignancies [7]. This is paired with general-purpose medical vision models, such as PanDerm, which outperformed certified clinicians by 10.2% in early-stage melanoma identification and enhanced non-dermatologist diagnostic capabilities by 16.5% across 128 diverse skin conditions [8]. Computer vision is also moving beyond 2D sections; advanced workflows utilize non-destructive 3D pathology to image the entire surface of an intact surgical specimen, eliminating the sampling errors inherent to traditional vertical cross-sections [9].

## 5. Molecular Biology as a Complementary Diagnostic Layer: Next-Generation Sequencing (NGS) and Gene Expression Profiling (GEP)

While computational intelligence proves highly effective at sorting patterns and calculating mathematical cell metrics, it is permanently restricted by visual architecture. If an underlying malignancy is morphologically silent or mimics an entirely indolent condition, pure vision-based algorithms hit the exact same morphological wall as the clinician. In atypical melanocytic proliferations, our specialty has seen a dramatic rise in descriptive categories reflecting this diagnostic uncertainty: Melanocytic Tumors of Uncertain Malignant Potential (MELTUMP), Superficial Atypical Melanocytic Proliferations of Unknown Significance (SAMPUS), Melanocytic Acral Nevus with Intraepidermal Ascent of Cells (MANIAC), De Novo Intraepidermal Epithelioid Melanocytic Dysplasia (DNIEMD), and Thin Melanoma of Uncertain Metastatic Potential (THIMUMP). To improve diagnostic resolution in these areas of morphological uncertainty, the digital ecosystem can be complemented by molecular data providing an additional biological layer of information.

### 5.1. Index Case Analysis

This multi-omic synergy is perfectly demonstrated by an index case study from the University Hospital of Geneva. A 45-year-old male presented with a changing, papular lesion on his right arm. The clinical differential diagnosis included seborrheic keratosis, a common melanocytic nevus, or verruca vulgaris.

**Figure 4:**
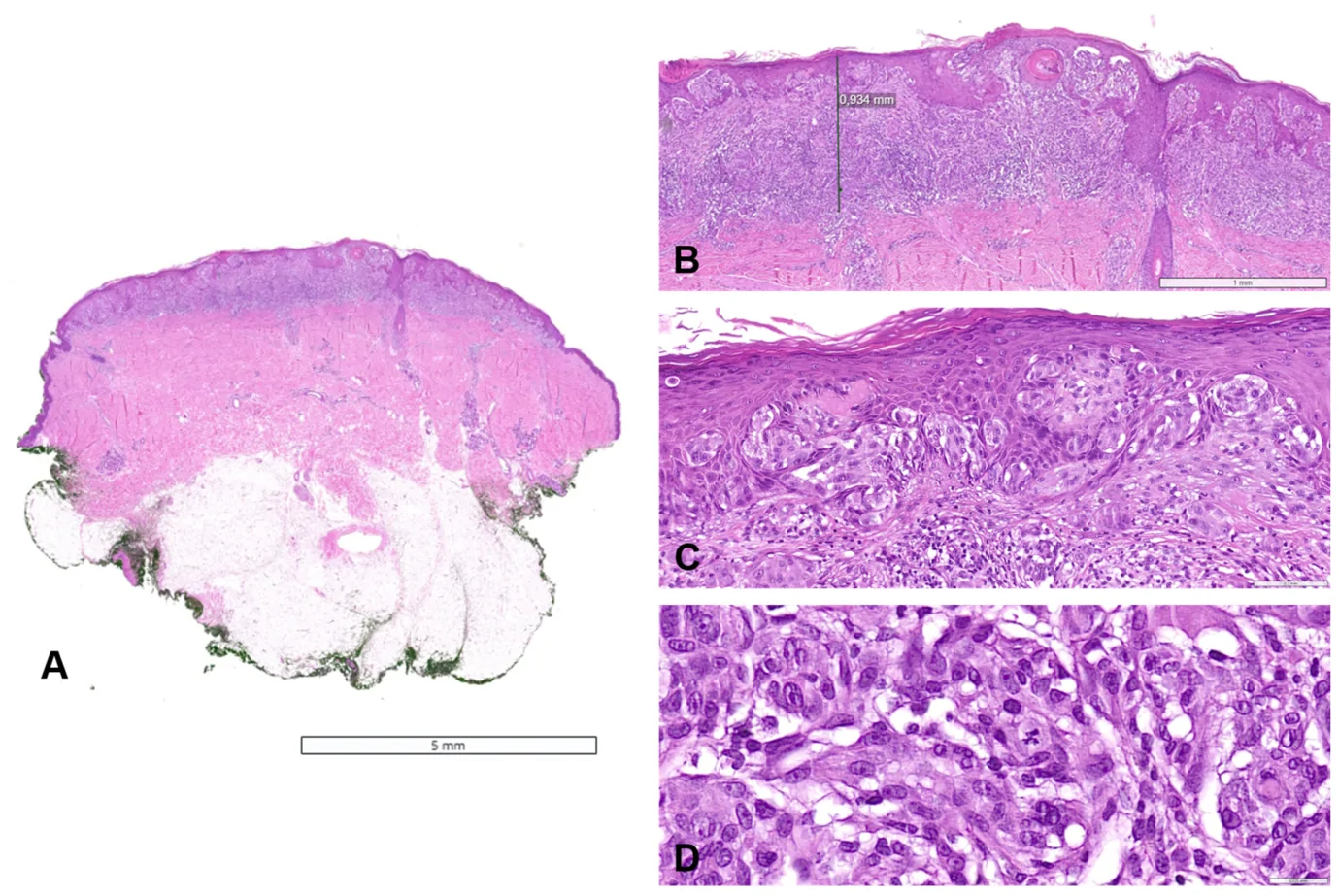
Histopathological examination showed a highly atypical intraepidermal and dermal melanocytic proliferation displaying spitzoid features associated with a dense, non-brisk inflammatory infiltrate and prominent pigment incontinence (**A**) (Scale bar: 5 mm). The dermal component extended into the mid-dermis over a measured thickness of 0.9 mm (**B**) (Scale bar: 1 mm). There were solitary melanocytes showing pagetoid spread (**C**) (Scale bar: 0.1 mm) with a mitotic index quantified at 2 mitoses/mm^2^ (**D**) (Scale bar: 0.025 mm).

Following complete surgical excision, histopathological examination at high magnification demonstrated a highly atypical melanocytic proliferation displaying prominent spitzoid features. The junctional component was situated within an acanthotic epidermis, characterized by vertically oriented cell nests containing large epithelioid and spindle cells with marked to severe cytologic atypia. Prominent Kamino bodies were identified, and significant trans-epidermal migration of cell nests and isolated melanocytes was observed. The dermal component extended into the mid-dermis over a measured thickness of 0.9 mm. The mitotic index was quantified at 2 mitoses/mm^2^, associated with a dense, non-brisk inflammatory infiltrate and prominent pigment incontinence (Figure 4).

**Figure 5:**
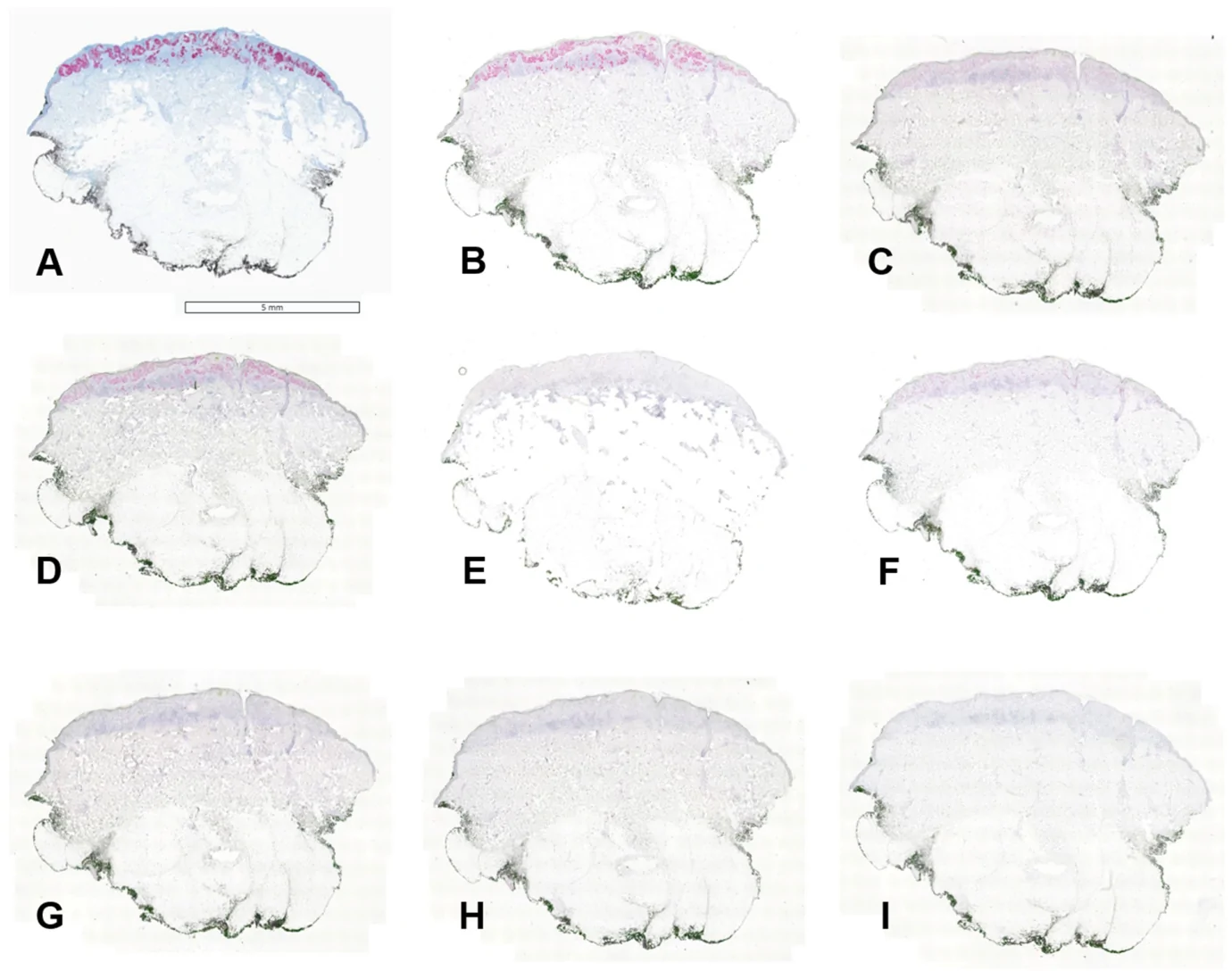
Immunohistochemical analysis showed strong diffuse positivity of Melan-A (**A**), SOX10 (**B**), and PRAME (**C**) in both the junctional and dermal components, confirming diffuse intra-epidermal ascent. HMB-45 demonstrated a complete absence of the expected maturation gradient toward the deeper portion of the lesion (**D**). BRAF V600 was negative (**E**). The Ki-67 (Mib-1) proliferation index was estimated at 10% in hot spots (**F**). ALK (**G**), NTRK (**H**) and ROS1 (**I**) were negative. (Scale bar: 5 mm).

A comprehensive immunohistochemical (IHC) panel was performed. Melan-A, SOX10, and PRAME demonstrated strong diffuse positivity in both the junctional and dermal components, confirming diffuse intra-epidermal ascent. However, HMB-45 demonstrated a complete absence of the expected maturation gradient toward the deeper portion of the tumor. The Ki-67 (Mib-1) proliferation index was estimated at 10% in hot spots, p16 expression was fully preserved, and programmed death-ligand 1 (PD-L1) expression demonstrated a Tumor Proportion Score (TPS) of 10%. Standard screening markers for common translocations, including ALK, ROS1, and NTRK, were entirely negative, and immunohistochemistry for mutated BRAF V600 was negative. At this specific stage, morphology and classic IHC profiles established a diagnosis of melanoma but failed to resolve the critical boundary defined by the 5th edition of the World Health Organization (WHO) classification [10]: a true Spitz melanoma versus a conventional melanoma displaying spitzoid features (spitzoid melanoma) (Figure 5).

### 5.2. Molecular Resolution and Genomic Classification

To achieve definitive diagnostic resolution, high-throughput Next-Generation Sequencing (NGS) was performed on extracted tumor RNA using an Illumina NextSeq platform and the TruSight RNA Fusion Panel. The analysis generated 63 million aligned reads and achieved an excellent technical quality score. Bypassing the visual ambiguity of the slide, the genomic analysis identified a highly specific, rare chromosomal translocation: an oncogenic *OPTN::RET* fusion linking exon 12 of *OPTN* (ENST00000263036) to exon 12 of *RET* (ENST00000355710) with an expression metric of 6.8 reads per million. Concurrent custom DNA-target hotspot capture (NGS100v2) confirmed a completely wild-type status for *BRAF*, *NRAS*, *KIT*, *GNAQ*, and *GNA11*.

According to recent studies by Donati et al. [11], Garbe et al. [12] and Quan et al. [13], true Spitz melanoma is characteristically driven by specific kinase alterations—including fusions in *ALK* (1%), *NTRK1/3* (2–5%), *ROS1* (1–2%), *MAP3K8* (9–33%), *BRAF* (3–8%) or *RET* (1–3%). Conversely, conventional spitzoid melanoma represents an ordinary sun-damaged tumor driven by conventional *BRAF* or *NRAS* mutations that merely mimics a Spitz tumor morphologically. The discovery of the *OPTN::RET* translocation definitively provided the final diagnosis of Spitz melanoma.

### 5.3. The GEP Crossroads and Therapeutic Mapping

Following diagnostic finalization, managing a Breslow 0.9 mm Spitz melanoma requires clear prognostic risk stratification to navigate clinical management regarding a Sentinel Lymph Node Biopsy (SLNB). In this context, bulk Gene Expression Profiling (GEP) via 31-GEP mRNA analysis represents an emerging adjunctive prognostic tool whose clinical utility and role in clinical management continue to evolve. This objective approach is supported by a large-scale meta-analysis of 1479 patients published by Greenhaw et al. [14] who demonstrated that GEP molecular classification significantly enhances standard AJCC staging. By combining anatomical staging (SLNB) with molecular biology (GEP), clinicians can manage discordant scenarios to prevent over-treatment or under-monitoring. These scenarios are illustrative/hypothetical rather than actual results obtained in the index patient.

- **Scenario A (Class 1A—Favorable Biology):** Backed by the landmark SEER registry collaboration data by Bailey et al. [15], a Class 1A profile indicates low risk carrying an exceptionally high Negative Predictive Value for regional metastasis and showing a 5-year distant metastasis-free survival rate of 94.1%. If the subsequent SLNB is negative, the clinical team achieves maximum reassurance; both anatomy and biology confirm low risk, fully justifying conservative wide local excision and standard clinical observation. If the SLNB is positive, it signals occult nodal spread (Stage III), but the underlying Class 1A signature suggests a potentially less aggressive, more indolent clinical course over time.
- **Scenario B (Class 1B/2A—Intermediate Biology):** A Class 1B or 2A profile represents an intermediate-risk biology, capturing patients who fall into a clinical gray zone between low and high metastatic potential. If the subsequent SLNB is negative, the team cannot rely on absolute reassurance; the intermediate signature necessitates a personalized, case-by-case monitoring strategy rather than standardized conservative follow-up. Conversely, a positive SLNB in this category confirms regional progression, requiring close multi-disciplinary tracking to determine the optimal threshold for systemic intervention.
- **Scenario C (Class 2B—Aggressive Biology):** A Class 2B profile indicates high metastatic risk and mortality, carrying a staggering Hazard Ratio of 7.00. If the SLNB is positive, it confirms an aggressive phenotype with active dissemination, triggering immediate escalation to medical oncology for systemic adjuvant therapy. However, if the SLNB is negative, the team enters “the critical trap.” Anatomical staging provides a false sense of security, but the high-risk molecular signature indicates that a significant risk of distant metastasis remains, demanding intensive radiographic surveillance despite “clear” regional lymph nodes.

Importantly, this multi-omic mapping delivers a potential therapeutic opportunity. If a high-risk patient eventually experiences systemic disease, the OPTN::RET fusion is no longer just a diagnostic marker—it represents an active genomic target. Depending on the available clinical evidence and a multidisciplinary assessment, the oncology team can consider deploying highly selective, small-molecule RET inhibitors such as Selpercatinib or Pralsetinib. These agents target downstream MAPK and PI3K signaling cascades to optimize the management of advanced disease.

Looking further toward the year 2030, our specialty must move past the tissue-averaging limits of bulk RNA assays, which homogenize tissue and erase intra-tumoral variations. The true future is High-Plex In Situ Spatial Transcriptomics. As demonstrated in pioneering methodology by Ståhl et al. [16] and advanced single-cell melanoma maps by Love et al. [17], spatial transcriptomics uses barcoded oligo probes to simultaneously profile 300+ to 5000+ multiplexed RNA targets directly on an FFPE section. This technology allows us to merge morphology and deep genomics onto a single digital canvas, verifying whether an oncogenic driver like *RET* is uniformly distributed throughout the lesion or confined to an aggressive subclone at the deep invasive front.

## 6. Current Challenges: Navigating the Gartner Hype Cycle

As dermatopathology stands at this digital and molecular crossroads, the field is tracking along the classic Gartner Hype Cycle. While older innovations like immunohistochemistry and basic molecular biology have reached the stable “plateau of productivity,” artificial intelligence is currently navigating the “peak of inflated expectations,” rapidly transitioning toward the “trough of disillusionment” as clinical labs confront real-world deployment realities. As demonstrated in a comparative evaluation by Alvarez-Silbergberg et al. [18], general-purpose conversational chatbots like ChatGPT and Gemini exhibit variable precision in interpreting oral pathology data, highlighting the limitations of using non-specialized large language models, underlining that these commercial tools are not substitutes for purpose-built diagnostic AI algorithms in complex clinical workflows.

To successfully operationalize these innovations, the medical community should confront five core challenges:

- Costs and Equity: High-capacity scanners, computational software licenses, and multi-omic panels carry immense capital costs, risking a system where precision medicine becomes an exclusive luxury.
- Ethics and Liability: If a sophisticated deep learning algorithm misclassifies a spitzoid melanoma as a benign Spitz nevus, the legal and ethical liability boundaries between the pathologist, the hospital, and the software developer remain entirely undefined.
- Lack of Diverse Perspectives: AI models trained on homogenous datasets introduce profound regional bias. Algorithms must be validated across diverse geographic cohorts and varied skin phototypes to prevent systemic diagnostic errors.
- External Quality Control Experts: WSI requires rigorous, standardized, external quality control protocols to manage tissue processing variations, slide artifacts, scanner calibration shifts, and digital color fidelity across different institutional networks.
- Human-Driven Medical Progress at Risk: Over-reliance on diagnostic automation and generative report writing threatens the fundamental training of junior residents. If the next generation of pathologists loses the ability to reason critically from first principles, human-driven medical discovery will be in danger.

## 7. Conclusions

The rapid convergence of digital primary workflows, artificial intelligence co-pilots, and advanced multi-omic profiling does not signal the disappearance of classical dermatopathology. Ultimately, the defining question for our generation is not whether AI will replace the pathologist, but rather how much it can augment our work and what kind of experts we must become.

The fear of machine replacement only exists if we narrow the definition of our profession to basic, visual pattern recognition. If our job description is simply “identifying shapes,” then we will eventually lose ground to automation. However, if we define our role as biological diagnostic specialists, technologies like WSI, NGS, GEP, and spatial transcriptomics do not replace us—they empower us. Morphology remains the absolute foundation; molecular biology provides the precise refinement; and multi-disciplinary collaboration is the definitive future.

The dermatopathologist of 2030 stands at the center of the clinical workflow, serving as the essential data integrator who translates complex visual architecture and high-dimensional genomics into meaningful diagnostic insight. Supported by unprecedented computational power, the dermatopathologist will remain responsible for the tasks that no algorithm can fully assume: interpreting context, navigating uncertainty, exercising clinical judgment, and making patient-centered decisions.

## Data Availability

No new data were created or analyzed in this study. Data sharing is not applicable to this article.
